# Supplementary material for: Change in childbearing intention, use of contraception, unwanted pregnancies, and related adverse events during the COVID-19 pandemic: Results from a panel study in rural Burkina Faso
Source: PLOS Glob Public Health. 2022 Apr 27;2(4):e0000174. doi: 10.1371/journal.pgph.0000174 (PMC10021617; doi:10.1371/journal.pgph.0000174)
Supplement: S1 Text — (DOCX) [file pgph.0000174.s001.docx]

Supporting information

**S1 Text Questions regarding knowledge and perceptions towards COVID-19**

Have you heard of COVID-19 / Coronavirus?

Yes

No

Don't know / refuse to answer

From what you have heard, are there any symptoms associated with COVID-19 / Coronavirus? If yes, which ones? (check all that apply)

Loss of smell or taste

Cough

Fever

Difficulty breathing

Chills

Sore throat

Diarrhea

Headache

Eye pain

Skin rash (redness, pimples)

There are no symptoms

Other

Don't know / refuse to answer

From what you have heard, are there any practices ("barrier actions") to protect yourself from COVID 19 / Coronavirus? If yes, what are they? (check all that apply)

Physical distance of one meter

Wearing a mask (or visor)

Avoid handshakes or hugs

Coughing into the crook of the elbow

Regular hand washing with soap or hydro-alcoholic gel

Avoid spitting on the ground

Avoid gatherings

Avoid touching your eyes, nose and mouth

Containment

No barrier gesture

Don't know / refuse to answer

Do you think that COVID19 / Coronavirus is a dangerous disease (for yourself)?

No

Yes, a little

Yes, a lot

Yes, very much

Don't know / refuse to answer

Do you think that COVID19 / Coronavirus is a dangerous disease for children?

No

Yes, a little

Yes, very much

Yes, very much

Don't know / refuse to answer

Do you have a hand washing facility in your household?

Yes, with soap

Yes, without soap

No

Don't know / refuse to answer

How many masks/face covers (disposable or reusable) do you have in your household today? (if none, mark 0; if does not know or refusal, mark 998)

Do you have a portable soap (hydro-alcoholic gel) that you can take with you?

Yes

No

Don't know / refuse to answer

Do you worry about catching COVID 19 when you go to a gathering place (e.g., church, market)?

No

Yes, a little

Yes, a lot

Yes, very much

Don't know

Are you afraid of catching COVID 19 when you go to a health center/hospital?

No

Yes, a little

Yes, a lot

Yes, very much

Don't know

Do you worry that anyone in your household (including yourself) will catch COVID 19 when a health care worker comes to give vaccines?

No

Yes, a little

Yes, a lot

Yes, very much

Don't know

Do you worry that a household member (including yourself) will catch COVID 19 when a health worker comes to give CPS?

No

Yes, a little

Yes, a lot

Yes, very much

Don't know

*Questions sur les connaissances et les perceptions à l’égard de la COVID-19*

|  | Avez-vous entendu parler de la COVID-19 / Coronavirus ? | Oui  Non  Ne sait pas / refuse de répondre |
| --- | --- | --- |
|  | D’après ce que vous avez entendu, y a-t-il des symptômes associés à la COVID-19 / Coronavirus ? Si oui, lesquels ?  (cochez tout ce qui s’applique) | Perte d'odorat ou de goût  Toux  Fièvre  Difficulté à respirer  Courbatures  Maux de gorge  Diarrhée  Maux de tête  Mal aux yeux  Éruption cutanée (rougeurs, boutons)  Il n’y a pas de symptômes  Autre  Ne sait pas / refuse de répondre |
|  | D’après ce que vous avez entendu, y a-t-il des pratiques (« gestes barrières ») pour se protéger de la COVID 19 / Coronavirus ? Si oui, lesquelles ?  (cochez tout ce qui s’applique) | Distanciation physique d'un mètre  Port du masque (ou visière)  Eviter les poignées de main ou embrassade  Tousser dans le creux du coude  Se laver régulièrement les mains avec du savon ou du gel hydro-alcoolique  Eviter de cracher par terre  Éviter les rassemblements  Eviter de se toucher les yeux, le nez, la bouche  Le confinement  Pas de geste barrière  Ne sait pas / refuse de répondre |
|  | Pensez-vous que la COVID19 / Coronavirus est une maladie dangereuse (pour vous-même) ? | Non  Oui, un peu  Oui, beaucoup  Oui, très fort  Ne sait pas / refuse de répondre |
|  | Pensez-vous que la COVID19 / Coronavirus est une maladie dangereuse pour les enfants ? | Non  Oui, un peu  Oui, beaucoup  Oui, très fort  Ne sait pas / refuse de répondre |
|  | Avez-vous un dispositif de lave-main dans votre ménage ? | Oui, avec du savon  Oui, sans savon  Non  Ne sait pas / refuse de répondre |
|  | Combien de masques / couvre-visages (jetables ou réutilisables) avez-vous dans votre ménage aujourd’hui ? | (si il n’y en a pas, indiquez 0 ; si NSP ou refus, indiquez 998) |
|  | Disposez-vous d’un savon portable (gel hydro-alcoolique) que vous pouvez emmener ? | Oui  Non  Ne sait pas / refuse de répondre |
|  | Est-ce vous craignez d'attraper la COVID 19 lorsque vous allez dans un endroit de rassemblement (par ex, l’église, le marché) ? | Non  Oui, un peu  Oui, beaucoup  Oui, très fort  Ne sait pas |
|  | Est-ce vous craignez d'attraper la COVID 19 lorsque vous allez dans un centre de santé / l’hôpital ? | Non  Oui, un peu  Oui, beaucoup  Oui, très fort  Ne sait pas |
|  | Est-ce que vous craignez qu'un membre de votre ménage (incluant vous-même) attrape la COVID 19 lorsqu’un membre du personnel de santé vient pour donner les vaccins ? | Non  Oui, un peu  Oui, beaucoup  Oui, très fort  Ne sait pas |
|  | Est-ce que vous craignez qu'un membre du ménage (incluant vous-même) attrape la COVID 19 lorsqu’un membre du personnel de santé vient pour donner la CPS ? | Non  Oui, un peu  Oui, beaucoup  Oui, très fort  Ne sait pas |
