# Supplementary material for: Change in childbearing intention, use of contraception, unwanted pregnancies, and related adverse events during the COVID-19 pandemic: Results from a panel study in rural Burkina Faso
Source: PLOS Glob Public Health. 2022 Apr 27;2(4):e0000174. doi: 10.1371/journal.pgph.0000174 (PMC10021617; doi:10.1371/journal.pgph.0000174)
Supplement: Attachment — Submitted filename: Response to reviewers.docx [file pgph.0000174.s002.docx]

Response to Reviewers

We would like to thank the editorial office and the reviewers for their comments. Please find below a point-by-point answer.

**Editorial Comments**

1. Please include additional information regarding the survey or questionnaire used in the study and ensure that you have provided sufficient details that others could replicate the analyses. For instance, if you developed a questionnaire as part of this study and it is not under a copyright more restrictive than CC-BY, please include a copy, in both the original language and English, as Supporting Information.

- The paragraph in the methods section has been modified to clearly indicate where the reader can find the questionnaire.

*A standardized sociodemographic questionnaire was administered individually to all participants. Questions extracted from the DHS instruments gathered data on household features (e.g., size, location, type of construction, possessions, etc.) which were used to create a socioeconomic index as per the DHS guidelines^29^. Using the DHS standardized questions, women were also asked about their reproductive health: history of live births and miscarriages, pregnancy status, childbearing intentions, and use of contraceptives (available at:* [*https://dhsprogram.com/publications/publication-dhsq7-dhs-questionnaires-and-manuals.cfm*](https://dhsprogram.com/publications/publication-dhsq7-dhs-questionnaires-and-manuals.cfm)*). Questions regarding knowledge and perceptions towards COVID-19 were added for the 2021 round (Additional file 1).*

2. You indicated that you had ethical approval for your study. In your Methods section, please ensure you have also stated whether you obtained consent from parents or guardians of the minors included in the study or whether the research ethics committee or IRB specifically waived the need for their consent.

- We added the information in the methods section.

*Participants aged 15-17 years old were considered mature minors and consented as adults.*

3. In the Methods, please clarify that participants provided oral consent. Please also state in the Methods:

- Why written consent could not be obtained

- Whether the Institutional Review Board (IRB) approved use of oral consent

- How oral consent was documented

For more information, please see our guidelines for human subjects research: <https://journals.plos.org/plosone/s/submission-guidelines#loc-human-subjects-research>

- Participants recruited in 2020 provided written consent. As requested by the IRBs, participants recruited in 2021 provided verbal consent. We modified the text in the methods section to add the requested information.

*All participants recruited in 2020 provided written informed consent. As suggested and approved by the research ethics committees, all participants recruited in 2021 provided informed verbal consent, in order to reduce risk of COVID-19 transmission. Verbal consent was recorded by the surveyor on the mobile data collection platform. The questionnaire was administered individually in a secluded area to preserve participant confidentiality. All the study procedures, including those for obtaining consent, were approved by the Comité d’éthique de la recherche en sciences de la santé at University of Montreal (Certificate #CERSES-20-146-D) and by the Comité d’éthique pour la Recherche en Santé in Burkina Faso (Deliberation #2018-6-075).*

4. Please ensure you have thoroughly discussed any potential limitations of this study within the Discussion section, including the potential impact of confounding factors.

- We added some information about potential limitations of this study.

*While the possibility of unobserved time-varying factors affecting the results cannot be ruled out, this evaluation design is one of the most rigorous in the absence of a control group43,44. Unfortunately, without a control group, it is impossible to estimate (nor to control for) what would have been the “normal trend” between 2020 and 2021.*

5. In the online submission form, you indicated that "All anonymized data used for this analysis can be made available by contacting the corresponding author under reasonable request.". All PLOS journals now require all data underlying the findings described in their manuscript to be freely available to other researchers, either 1. In a public repository, 2. Within the manuscript itself, or 3. Uploaded as supplementary information.

This policy applies to all data except where public deposition would breach compliance with the protocol approved by your research ethics board. If your data cannot be made publicly available for ethical or legal reasons (e.g., public availability would compromise patient privacy), please explain your reasons by return email and your exemption request will be escalated to the editor for approval. Your exemption request will be handled independently and will not hold up the peer review process, but will need to be resolved should your manuscript be accepted for publication. One of the Editorial team will then be in touch if there are any issues.

- We request an exemption. As stipulated by the IRBs, the data cannot be used for secondary analysis, which is impossible to guarantee if the dataset is uploaded to a public repository with unrestricted use.

6. Please provide us with a direct link to the base layer of the map used in Figure 1 and ensure this location is also included in the figure legend.

Please note that, because all PLOS articles are published under a CC BY license (creativecommons.org/licenses/by/4.0/), we cannot publish proprietary maps such as Google Maps, Mapquest or other copyrighted maps. If your map was obtained from a copyrighted source please amend the figure so that the base map used is from an openly available source.

Please note that only the following CC BY licences are compatible with PLOS licence: CC BY 4.0, CC BY 2.0 and CC BY 3.0, meanwhile such licences as CC BY-ND 3.0 and others are not compatible due to additional restrictions. If you are unsure whether you can use a map or not, please do reach out and we will be able to help you.

The following websites are good examples of where you can source open access or public domain maps:

* U.S. Geological Survey (USGS) - All maps are in the public domain. (http://www.usgs.gov)

* PlaniGlobe - All maps are published under a Creative Commons license so please cite “PlaniGlobe, http://www.planiglobe.com, CC BY 2.0” in the image credit after the caption. (http://www.planiglobe.com/?lang=enl)

* Natural Earth - All maps are public domain. (<http://www.naturalearthdata.com/about/terms-of-use/>)

- We added the requested information.

*Data and layers used to create this map are available at:* [*https://data.humdata.org/dataset/burkina-faso-administrative-boundaries*](https://data.humdata.org/dataset/burkina-faso-administrative-boundaries)

7. Please ensure that the funders and grant numbers match between the Financial Disclosure field and the Funding Information tab in your submission form. Note that the funders must be provided in the same order in both places as well. Currently, MS holds a Tier 2 Canada Research Chair in Urban Health Equity and Young People information is not included in the Funding Information.

- We added the information in the Funding Information tab in the submission form.

**Reviewer #1**

The manuscript addresses the relevant issue of reproductive health in the context of LMIC's in times of COVID19. The text is clear and well written, as is the methodological design. The results are quite interesting, but presented in a way that seems inappropriate, as follows:

Table 1: Does not inform the population of women to whom the data refer to: if all women of reproductive age (N=814 + 87; 597 + 64), or if only sexually active women (N=814; 597 ), since the totals of the different variables show inconsistencies. In addition, comparative data on the 540 women who were actually surveyed at both times (2020 and 2021) should be presented. The title also lacks place and years, and does not clarify that the time lapse is 12 months.

- We modified the table 1 accordingly.

Figure 2: The title lacks to indicate the effect measure, the sampled population and the study site. The text mentions adjusted OR, but does not say for which variables, while the figure suggests crude OR.

- We modified the title of Figure 2, and we substituted the expression “adjusted odds ratio” by “odds ratio with fixed effects” in the text (legend).

Figure 3: The x-axis is missing. Text mentions about significant and non-significant results, but the levels of significance are not presented. It is not clear whether the texts referring to figures 2 and 3 (lines 284-285 and 288-292) are comments or footnotes.

- We added the x axis in Figure 3. We added the levels of significance in the text. Lines 284-285 and 288-292 are footnotes; PLoS GPH requires that legends of the figures are placed within the text. The confusion should be fixed in the final proof.

Figure 4: The level of significance of the difference between the curves is not shown. Title is also inappropriate.

- We modified the title. Figure 4 does not present tests of significance between the curves, but rather the 95% confidence interval surrounding the predicted probabilities of each outcome, by age. Each curve is therefore surrounded by the corresponding 95% confidence interval; we explained this in the legend. Results of tests of significance between the mean differences are presented in Table 2.

Table 2 and 3: Similar formatting issues, already mentioned.

- Tables 2 and 3 were modified accordingly.

Finally, the results of the analyses, although coming from different regression models, should be presented in tables with a basic standardization. The figures should only serve to illustrate the results in the tables.

- In order to standardize the results, we produced a single type of effect measure, i.e. risk ratios. We carefully reviewed the format of all tables and figures. All data supporting the interpretation of the figures are either presented in tables or in the text.

The discussion seemed appropriate. However, a possible limitation of the study would be that the date of the last birth and the occurrence of miscarriages, abortions and stillbirths were not evaluated as potential confounding factors in the association between the desired lapse of time until the next pregnancy and the COVID19 pandemic.

- It is not recommended to control for variables that were potentially affected by the intervention – including these as confounding variables would have led to increase the risk of collider bias. However, we agree that the effect of COVID19 could have been mediated by these variables. We include this in the limitation.

**Reviewer #2**

I would like to suggest that the authors explain how they arrived at the number of 700 households, based on which parameters, which epidemiological criteria for calculating the sample size of women of childbearing age, in the methods. Was it based on the prevalence of contraceptive use, for example?

- Unfortunately, the sample size was not calculated for the purpose of this particular study, since it was a natural experiment provoked by the pandemic situation. The sample size was originally decided for the purpose of another study. We added this information in the methods section, and discuss it as a limitation of the present study.

*All study parameters were originally decided based on the SYNERGIE study protocol; the present study is a natural experiment that exploited the already existent research platform to evaluate the effects of an unanticipated event, i.e. the occurrence of the pandemic.*

Furthermore, in lines 209-210 – authors wrote that interviewers were trained for qualitative research, but the study is quantitative.

- This was an error. We replaced by “epidemiological research”.

In lines 341-343, the authors discussed measures taken locally to expand the supply of contraceptives in this population. But I did not find data on the birth rate, which would be interesting to contextualize how the health system works in this area to assist family planning.

- We added some information about the trends in birth rate and unmet needs for family planning in Burkina Faso using the most recent data.

*The upward trend in contraceptive use has been steady and rapid since 2015; that year, the contraceptive prevalence rates in the Cascades and Centre-Ouest regions (where the study area is located) were estimated to be 21% and 28%, respectively, while the national prevalence of unmet need for family planning reached 35%. The World Bank estimates suggest a gradual decline in the birth rate in Burkina Faso over the past decade, but it remains one of the highest in the world, with 37 births per 1,000 inhabitants per year in 2019.*
